# Tapered self-expandable metallic stent optimized for Eustachian tube morphology in a porcine ET model

**DOI:** 10.1038/s41598-022-24615-6

**Published:** 2022-11-24

**Authors:** Jeon Min Kang, Song Hee Kim, Dae Sung Ryu, Yubeen Park, Dong-Sung Won, Ji Won Kim, Chu Hui Zeng, Woo Seok Kang, Jung-Hoon Park, Hong Ju Park

**Affiliations:** 1 Biomedical Engineering Research Center, Asan Institute for Life Sciences, Asan Medical Center, 88 Olympic-Ro 43-Gil, Songpa-Gu, Seoul, 05505 Republic of Korea; 2 Department of Otorhinolaryngology-Head and Neck Surgery, Asan Medical Center, University of Ulsan College of Medicine, 88 Olympic-Ro 43-Gil, Songpa-Gu, Seoul, 05505 Republic of Korea

**Keywords:** Biomedical engineering, Preclinical research, Translational research, Anatomy

## Abstract

Several investigations on the feasibility of stent placement into the Eustachian tube (ET) are being conducted. However, stents optimized for the anatomical structure of the ET have not yet been developed. In this study, the efficacy and safety of a self-expandable metallic stent (SEMS) optimized for porcine ET morphology was investigated. Silicone was injected into a cadaveric porcine ET to analyze the ET morphology. The three-dimensional-reconstructed porcine ET phantom images obtained after a computed tomography scan were measured to determine the dimensions of the porcine ET. The SEMS was designed as a tapered structure on the basis of the morphological findings of the porcine ET. The tapered SEMS (T-SEMS) and conventional SEMS (C-SEMS) were placed into the porcine ET to compare the safety and efficacy of the two types of SEMSs. Stent-induced tissue hyperplasia in the T-SEMS group was significantly lower than that in the C-SEMS group (*p* < 0.001). The T-SEMS optimized for the porcine ET was effective in maintaining stent patency. T-SEMS seems to be better than C-SEMS in suppressing stent-induced tissue hyperplasia, owing to the reduced stent-mediated mechanical injuries and maintaining ET patency.

## Introduction

The Eustachian tube (ET) is an organ connecting the middle ear cavity to the nasopharynx that performs various functions in the middle ear, including providing ventilation, protection against pathogenic microorganisms, and drainage of secretion to the nasopharynx^1^. Unlike other non-vascular luminal organs, the ET has a conical shape that consists of two portions^2,3^. The first portion, distal one-third of the ET, is narrow and surrounded by bony structures. Toward the nasopharynx, the second portion of the ET becomes wider and is called the cartilaginous portion. When the cartilaginous portion does not open or close properly, ET dysfunction (ETD) occurs, which has the potential to become obstructive^4–6^.

Various therapeutic strategies, such as laser Eustachian tuboplasty, ventilation tube insertion, microdebrider tuboplasty, and balloon Eustachian tuboplasty (BET), can be applied for the treatment of ETD^7–9^. In particular, BET, which uses a balloon catheter, has emerged as a minimally invasive intervention for the treatment of ETD^10^. The clinical success rate of the BET was reported between 36 and 80%. Some patients fail to respond to balloon dilatation, and the effects of BET slowly decrease over time^10–12^. Resistant cases of ETD requiring repeated BET have a major negative impact on the quality of life. Thus, finding other effective therapeutic options remains a therapeutic challenge. Previous studies proposed the use of ET stenting as an alternative option for patients with ETD, and preclinical studies were conducted to develop an ET stent for relieving ETD^13–18^. However, the tubular structure of the ET stents used in the previous studies may not be appropriate for the morphology of the ET. In this study, the morphology of the porcine ET was analyzed based on a computed tomography (CT)-scanned porcine ET phantom. An ET stent made of self-expanding nitinol wire was designed and manufactured on the basis of the findings of the ET morphology. Thus, this study aims to investigate the efficacy and safety of a tapered self-expandable metallic stent (T-SEMS) optimized for porcine ET morphology in comparison with a conventional SEMS (C-SEMS) in porcine ET.

## Materials and methods

### Morphological analysis of cadaveric porcine ET

**Figure 1:**
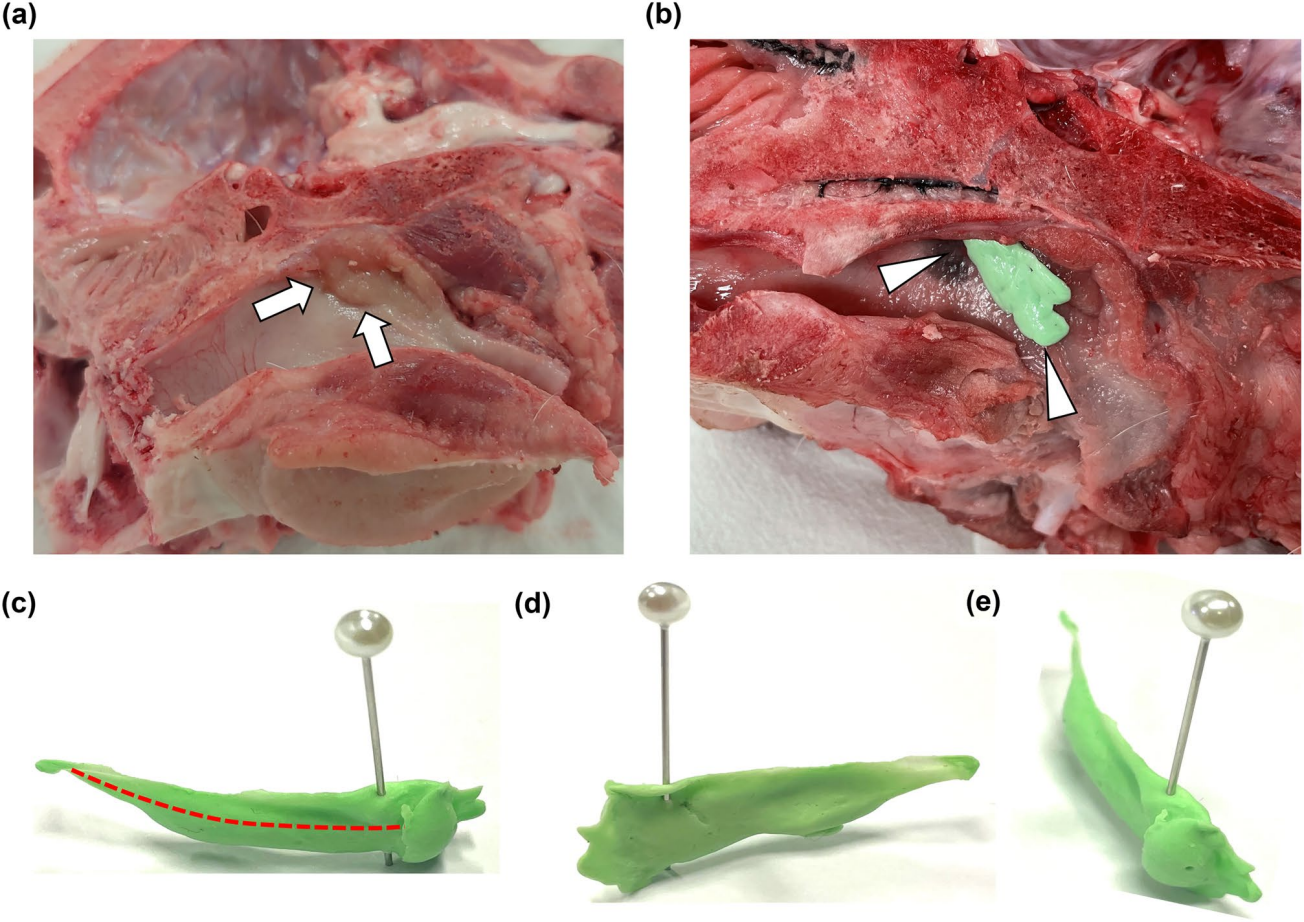
Fabrication of the cadaveric porcine ET phantom. (**a**) The nasopharyngeal ostium (*arrows*) of the cadaveric porcine head was identified following mid-sagittal sectioning. (**b**) Silicone (*arrow heads*) was injected until completely filling the ET. (**c**,**d**) The two lateral and (**e**) anterior projections of the silicone phantom extracted from the porcine ET.

This experiment was approved by the Institutional Animal Care and Use Committee (IACUC) of the Asan Institute for Life Sciences and conformed to the ARRIVE guidelines for humane handling of laboratory animals (IACUC-2020-12-189). All the experiments were performed in accordance with relevant guidelines and regulations. Six ETs of three fresh cadaveric porcine heads (Yorkshire, 33.4–36.7 kg at 3 months; Orient Bio, Seongnam, Korea) were used in this study. The porcine heads were mid-sagittally sectioned to identify the nasopharyngeal ostium. A porcine ET phantom was fabricated using the fabrication process of a cadaveric ET phantom in a previous study^19^. A 100 μl pipette tip was connected to a 10 ml syringe, and the end tip of the pipette was inserted into the nasopharyngeal ostium. Silicone (Otoform AK®, Dreve Otoplastik GmbH, Unna, Germany) was injected through the pipette into the porcine ET until it completely covered the nasopharyngeal ostium, and constant pressure was then applied in the ET (Fig. 1). The silicone-filled porcine heads were kept at 4 °C for 12 h, and the cured silicone was later separated from the porcine ET.

### Image analysis of porcine ET phantom

A micro-CT imaging system (NanoPET/CT, Mediso Ltd–Bioscan Inc., Arlington, Texas, USA) was used to quantitatively analyze the size of the porcine ET phantom. CT images were reconstructed to analyze the morphology of the porcine ET phantoms. Three-dimensional-reconstructed images were obtained from the portions axially sectioned at 4 mm intervals from the nasopharyngeal ostium to the isthmus. A total of six sections were obtained every 4 mm, and points were defined from P_0_ to P_5_. The height of the axially sectioned ET was measured as the length from the top to bottom of the ET lumen. The width of the axially sectioned ET was measured as the maximum distance between the anterior and posterior sides. The overall length of the ET was measured as the distance from the nasopharyngeal ostium to isthmus.

### Stent preparation

**Figure 2:**
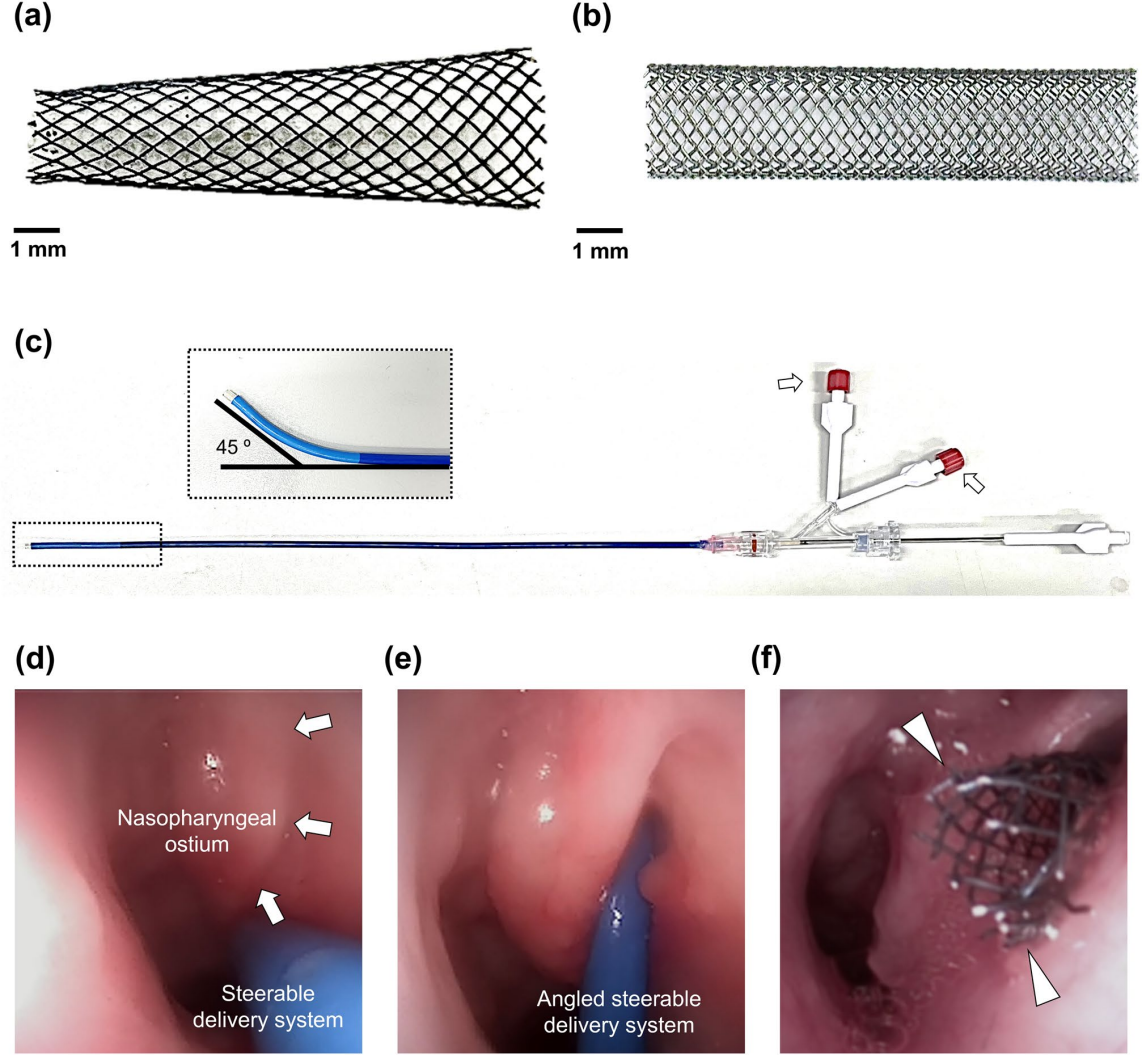
Self-expandable metallic stents (SEMSs) with the delivery system and the technical steps for stent placement into the porcine ET. Photographs showing (**a**) the tapered SEMS (T-SEMS) and (**b**) conventional SEMS (C-SEMS). (**c**) The steering control threads (*arrows*) were used to modulate the angle of the proximal end of the stent delivery system for easy access to ET orifice (maximum curved angle was 45°). (**d**) Endoscopic image showing the nasopharyngeal ostium (*arrows*) and inserted steerable delivery system. (**e**) The angled delivery system was inserted into the nasopharyngeal ostium. (**f**) The T-SEMS (*arrow heads*) was successfully placed into the ET.

All SEMSs (S&G Biotech Co., Ltd, Yongin, Korea) used in this study were designed and manufactured based on the morphological findings of the porcine ET phantom. A total of 32 nitinol wires with a thickness of 0.09 mm were interwoven using a braiding machine. The SEMS optimized for the porcine ET had a tapered structure. When fully expanded, the T-SEMS was 2 mm in diameter at the distal end and 5 mm in diameter at the proximal end of the T-SEMS and 16 mm in length (Fig. 2a). The C-SEMS had a tubular structure and was 3 mm in diameter and 16 mm in length (Fig. 2b).

### Steerable stent delivery system

The SEMS delivery system consisted of a 9 Fr steerable sheath (OSYMED. Co., Ltd, Yongin, Korea), steering control threads, and a pusher catheter (Fig. 2c). The maximum curved angle of the SEMS delivery system was 45°. The steerable SEMS delivery system was developed for easy access to the nasopharyngeal ostium of the porcine ET.

### Animal study

This part of the experiment was also approved by IACUC and conformed to the ARRIVE guidelines. A total of six ETs of three pigs (Yorkshire; Orient Bio) weighing 34.2–36.5 kg was divided into two groups that received the T-SEMS or C-SEMS. All pigs were supplied with water and food ad libitum and were maintained at a temperature of 24 ± 2 °C with a 12-h day–night cycle. Subsequently, all pigs were euthanized 4 weeks after the stent placement by intravenously injecting potassium chloride (DAI HAN PHARM CO., Seoul, Korea).

### Stent placement into the porcine ET and endoscopic examination

All pigs were anesthetized immediately before stent placement using a mixture of 50 mg/kg zolazepam, 50 mg/kg tiletamine (Zoletil 50; Virbac, Carros, France), and 10 mg/kg xylazine (Rompun; Bayer HealthCare, Leverkusen, Germany). An endotracheal tube was then placed, and anesthesia was administered by inhalation of 0.5–2% isoflurane (Ifran®; Hana Pharm. Co., Seoul, Korea) with 1:1 oxygen (510 ml/kg per min). Endoscopic examination (VISERA 4K UHD Rhinolaryngoscope; Olympus, Tokyo, Japan) was performed to check the nasopharyngeal ostium of the ET. The steerable delivery system loaded with the T-SEMS or C-SEMS was advanced through the nostril to the nasopharyngeal ostium of the ET under endoscopic guidance (Fig. 2d). The steerable delivery system was bent toward the nasopharyngeal orifice and carefully inserted into the ET until it met with resistance in the isthmus portion of the ET (Fig. 2e). The stent was placed into the ET by withdrawing the delivery system, while the pusher catheter in place (Fig. 2f). Post-procedure endoscopic examination was performed to evaluate any procedure-related complications and to determine the location of the proximal end of the stent. An endoscopic examination was conducted on the pigs at 4 weeks after the stent placement to evaluate the stent position, patency, and secretion presence around the stent.

### Histological examination

A histological examination was conducted on the basis of previous ET stent studies^14,15^. The stented ET tissues were extracted. The ET tissue samples were fixed in 10% neutral-buffered formalin for 3 days. After fixation, the samples were embedded in a resin block. The resin blocks were sliced from the proximal and distal portions of the segment using the grinding system (Apparatebau GmbH, Hamburg, Germany). The slides were stained with hematoxylin–eosin for the histological evaluation. The histological evaluations were performed to assess the percentage of stent-induced tissue hyperplasia and the degree of inflammatory cell infiltration. The percentage of tissue hyperplasia of ET was calculated using the following equation:

$$100 \times \left(1- \frac{Stenotic \, area \, of \, stent \, \left({\mathrm{mm}}^{2}\right)}{Original \, area \, of \, stent \, \left({\mathrm{mm}}^{2}\right)}\right).$$

The degree of inflammatory cell infiltration is determined based on the distribution and density of inflammatory cells. Measurement indicators 1, 2, 3, 4, and 5 indicate mild, mild to moderate, moderate, moderate to severe, and severe inflammatory cell infiltration, respectively^20^. Observations for the histological analysis of the ET were obtained using a microscope (BX51; Olympus, Tokyo, Japan), and measurements were made using the CaseViewer software (CaseViewer; 3D HISTECH Ltd., Budapest, Hungary). The histological findings were verified based on the consensus of three observers blinded to the study.

### Statistical analysis

The Mann–Whitney U test was used to analyze the differences between the groups as appropriate. A value of *p* < 0.05 was considered statistically significant. A Bonferroni-corrected Mann–Whitney U test was performed for *p* values < 0.05 to detect group differences (*p* < 0.008 as statistically significant). Statistical analyses were performed using the SPSS software (version 27.0; SPSS, IBM, Chicago, IL, USA).

### Ethics declarations

All experiments were performed in accordance with relevant ARRIVE guidelines and regulations.

### Ethical approval

All experiments were approved by the Institutional Animal Care and Use Committee of the Asan Institute for Life Sciences (IACUC-2020-12-189).

## Results

### Morphological findings of porcine ET

**Figure 3:**
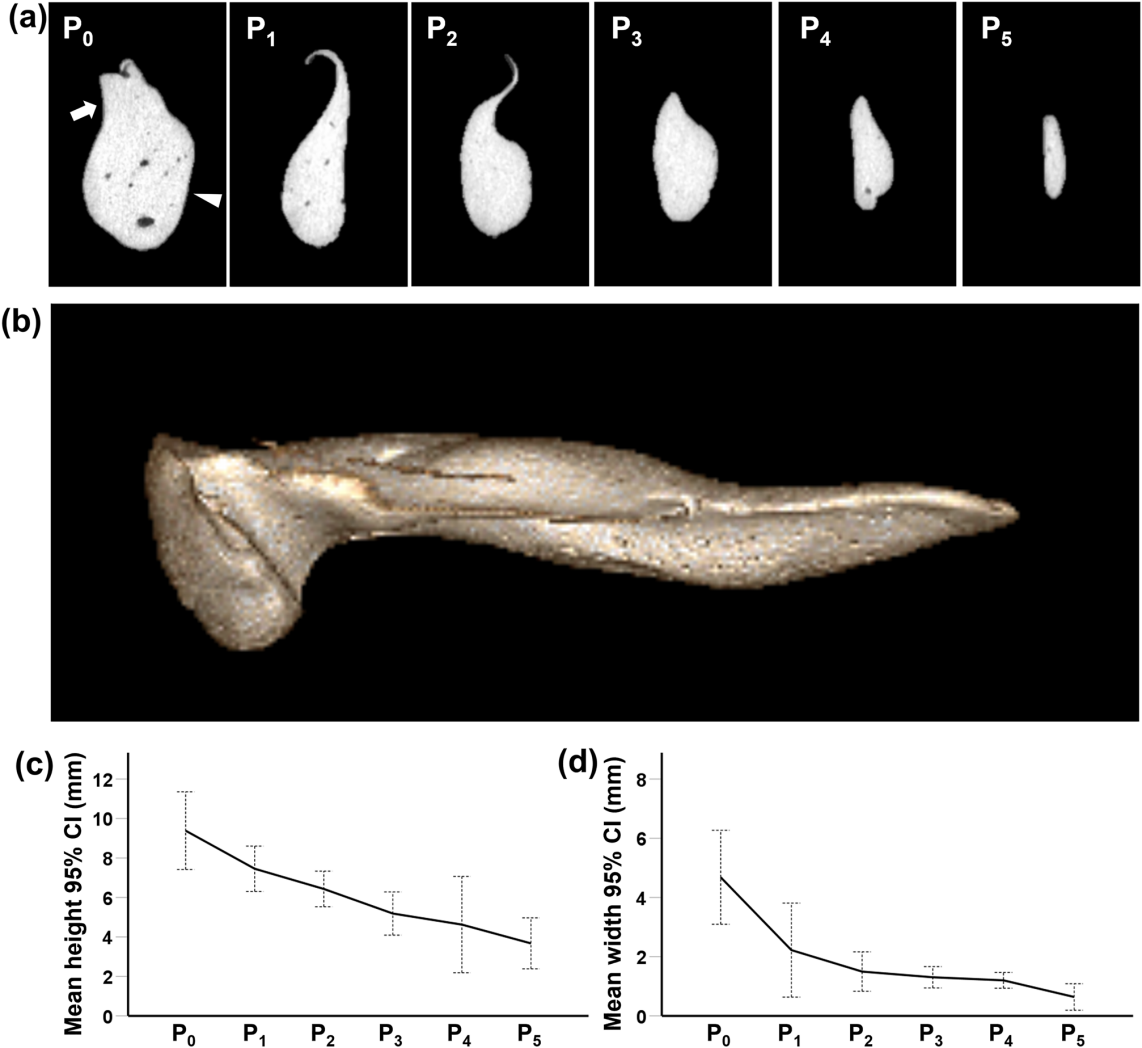
Morphological findings and measurements of the porcine ET phantom. (**a**) Axially sectioned CT images of the porcine ET phantom showing the dilated ET in a curved-cone shape. ET lumen consists of the Rüdinger safety canal (*arrow*) and the auxiliary gap (*arrowhead*). (**b**) 3D-reconstructed CT images of the porcine ET phantom showing a posterolateral curvature until midpoint, followed by a curved anterior wall. The (**c**) height and (**d**) width of the axially sectioned porcine ET phantom gradually decreased from the nasopharyngeal ostium to the isthmus.

The morphological findings are shown in Fig. 3. The porcine ET phantom was successfully fabricated without mucosal injuries to the lumen. The ET started from the nasopharyngeal ostium and initially showed a posterolateral curvature. From the midpoint to the isthmus, a slight anterior curvature was observed. The area of the nasopharyngeal ostium was the most widely expanded, and the ET lumen became smaller toward the isthmus. Inside the ET, the cross section had the shape of a quotation mark. The mean (± standard deviation; SD) overall length of the ET was 23.18 ± 2.94 mm. The mean (± SD) height of the axially sectioned porcine ET phantom gradually decreased from 9.39 ± 1.87 mm at P_0_ (nasopharyngeal ostium) to 3.67 ± 1.23 mm at P_5_ (isthmus). The mean (± SD) width also gradually decreased from 4.68 ± 1.51 mm at P_0_ to 1.20 ± 0.25 mm at P_4_ (the distal end of the cartilaginous portion). P_5_ (isthmus) was 0.64 ± 0.42 mm in width and significantly smaller than P_4_.

### Procedural outcomes and endoscopic findings

**Figure 4:**
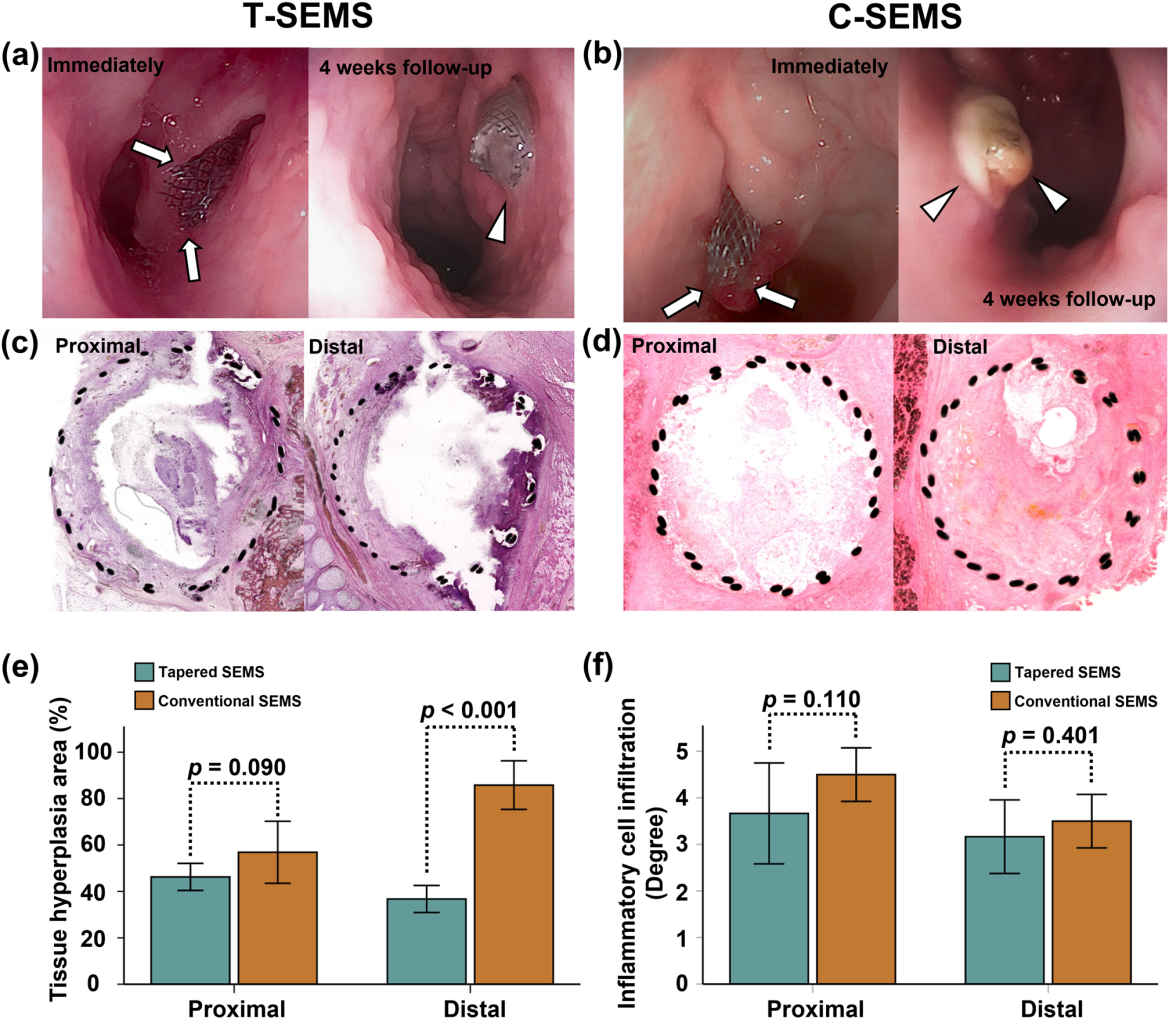
Representative endoscopic and histological images of the porcine ET. (**a**) Mild secretion (*arrow head*) around the T-SEMS (*arrows*) at 4 weeks. (**b**) Relatively severe secretion (*arrow heads*) was observed around the C-SEMS (*arrows*) at 4 weeks after the stent placement. (**c**,**d**) Histological images show significantly higher percentage of tissue hyperplasia in the distal portion of the C-SEMS group than that of the T-SEMS group. Histological results of the (**e**) percentage of tissue hyperplasia and (**f**) degree of inflammatory cell infiltration at 4 weeks after the stent placement in the T-SEMS and C-SEMS groups. *T-SEMS* tapered self-expandable metallic stent, *C-SEMS* conventional self-expandable metallic stent.

All SEMS placements were technically successful in the pigs without any procedure-related complications. The proximal ends of all the SEMSs were observed to be protruding from the ET in the endoscopic examination following the procedure. A follow-up study was conducted at 4 weeks, which showed no stent-related complications, such as stent migration, during the follow-up period. All SEMSs preserved their round shapes. However, a secretion around the proximal end of the SEMSs was observed in the endoscopic follow-up at 4 weeks (Fig. 4a,b). All pigs lived until the end of the study.

### Histological findings

The histological findings are shown in Fig. 4c–f. The mean (± SD) percentage of tissue hyperplasia in the proximal portion was not significantly different between the two groups (46.25 ± 5.56% in the T-SEMS group vs. 56.88 ± 12.73% in the C-SEMS group, *p* = 0.090). However, the distal portion in the T-SEMS group (36.73 ± 5.54%) was significantly lower than that in the C-SEMS group (85.82 ± 9.98%, *p* < 0.001). The degree of inflammatory cell infiltration was not significantly different in the proximal and distal portions between the two groups (proximal portion: 3.66 ± 1.03 in the T-SEMS group vs. 4.5 ± 0.54 in the C-SEMS group, *p* = 0.110 and distal portion: 3.16 ± 0.75 in the T-SEMS group vs. 3.5 ± 0.54 in the C-SEMS group, *p* = 0.401).

## Discussion

The anatomy of the ET is complex and narrow, which is different from other luminal organs^1^. Our study investigated the overall shape and dimensions of the porcine ET using the porcine ET phantom. A previous study reported the average total length of the ET as 24.2 mm using a cadaveric miniature porcine ET model^21^. This was close to the average length of the porcine ET phantom (23.18 mm) observed in our study. It was verified that the width of the ET decreased closer to the isthmus portion. In particular, the average width of the ET sharply decreased between the distal end of the cartilaginous portion and the isthmus (P_4_: 1.20 ± 0.25 mm vs. P_5_: 0.64 ± 0.42 mm). It is unclear whether ET dilation near the end of the isthmus portion is effective in treating ET dysfunction. Therefore, the length of the T-SEMS was determined to be 16 mm to fully cover the distal end of the cartilaginous portion of the ET.

The ET lumen consists of two compartments: the Rüdinger safety canal and auxiliary gap^22,23^. Below the Rüdinger safety canal, there is a second area called the auxiliary gap. The auxiliary gap repeatedly opens and closes with the movement of the surrounding muscles. The opening of the ET is restricted to this area. Therefore, the diameter of the T-SEMS is determined on the basis of the width of the ET lumen, which is the diameter of the auxiliary gap. The average width of the nasopharyngeal ostium was 4.68 ± 1.51 mm and that of the distal end of the cartilaginous portion was 1.20 ± 0.25 mm. Based on the morphological findings of the porcine ET, the T-SEMS was designed as a tapered structure with diameters of 5 mm and 2 mm at the proximal and distal ends of the SEMS, respectively.

Our results demonstrated that stent-induced tissue hyperplasia at 4 weeks after stent placement significantly decreased in the T-SEMS group compared with that in the C-SEMS group in the porcine ET. Wound healing by mechanical injuries resulting from stent placement is divided in early inflammatory, late proliferative, and tissue remodeling phases within 4 weeks^24–26^. Severe stent-induced tissue hyperplasia was evident at 4 weeks after stent placement in the porcine ET^13^. The tissue hyperplasia area was significantly different in the distal portion of the SEMSs between the two groups, whereas no significant difference was observed in the proximal portion of the SEMSs between the two groups. The T-SEMS exhibits less stent-mediated mechanical injuries in the distal portion than the C-SEMS. When a large diameter or strong radial force of the SEMS is used, stent-induced mechanical injuries increase, which can lead to the promotion of stent-induced tissue hyperplasia^27,28^. Placement of a C-SEMS of constant diameter in the tapering ET lumen may result in occlusion owing to stent oversizing in the distal portion. In addition, occlusion of the ET lumen leads to ETD, resulting in the accumulation of mucus in and around the ET^1,4,29^. The C-SEMS showed severe mucus accumulation, which was difficult to remove in the endoscopic findings and was similar to ETD. However, in the T-SEMS, a mild secretion was observed, which was easy to remove, indicating the increased possibility of the patent stent. These findings suggest that a stent with a tapered structure has high efficacy with regard to ET than a stent with a tubular structure.

The shape of the stent can also be altered by movements surrounding the ET in a living animal, such as swallowing and chewing^4,5^. Previous studies reported that the distal end of the stent collapsed because of the insertion of a cobalt–chrome stent^13,14,17^. Therefore, the T-SEMS and C-SEMS were fabricated with nitinol, a self-expandable metal. The self-expandable metal exhibits super elasticity, which can maintain a round shape even under external pressure. In this study, all stents maintained their round shape without stent collapse. However, there was no significant difference in the degree of inflammation around the stent struts. This may be because all the stents are made of the same material.

The feasibility of stenting in ET has been verified in many preclinical studies^13–18^. However, previous studies have been conducted with commercially available stents that are not optimized for ET. In a human cadaver study, stents with different diameters were inserted and evaluated; however, all stents were not fully dilated at the distal cartilaginous portion^18^. Additionally, a commercially available coronary cobalt–chrome stent was implanted in the ET of sheep and pigs and subsequently evaluated^13,14,17^. In this study, a T-SEMS made of self-expanding nitinol wire was optimized for the ET. Therefore, we believe that the therapeutic strategy of using T-SEMS to treat obstructive ET may prolong stent patency by suppressing stent-induced tissue hyperplasia, thus improving the quality of life of the patients.

Our study has some limitations. First, although there were significant differences in the statistical analysis, the number of animals in this study was small, and a robust statistical analysis could not be performed. Additional study with large number of animals is needed to enhance our findings. Second, tissue responses were observed after stenting in the normal porcine ET. The diameter and length of the stent can vary depending on the degree of stenosis of the ET. Finally, our study did not compare the mechanical properties between the T-SEMS and C-SEMS. The mechanical properties of the stent structure, including radial forces, axial tension, and torsional test results of the stent, must be evaluated. Although the mechanical properties of T-SEMS have not been evaluated, our results demonstrate the basic concept that the tapered structure of the SEMS is effective with respect to the anatomical structure of the ET.

## Conclusion

The T-SEMS, which was designed based on the morphological analysis of the porcine ET, was effective and safe with regard to maintaining stent patency. Additionally, it suppressed stent-induced tissue hyperplasia, owing to the reduced stent-mediated mechanical injuries compared with that in the C-SEMS in porcine ET. Although further preclinical studies are required to investigate SEMSs with respect to their mechanical properties and optimized shapes for the ET, our findings may serve as a basis for future investigations on the development of SEMS for the ET.

## Data Availability

All data generated or analyzed during this study are included in this published article.
